# Clinicopathologic characteristics and prognostic significance of HER2-low expression in patients with early breast cancer: A systematic review and meta-analysis

**DOI:** 10.3389/fonc.2023.1100332

**Published:** 2023-02-02

**Authors:** Tong Wei, Dingyuan Wang, Songlin Gao, Xue Wang, Jian Yue, Yikun Kang, Jie Ju, Zixuan Yang, You Shuai, Peng Yuan

**Affiliations:** ^1^ Department of VIP Medical, National Cancer Center/National Clinical Research Center for Cancer/Cancer Hospital, Chinese Academy of Medical Sciences and Peking Union Medical College, Beijing, China; ^2^ Department of Breast Surgery, National Cancer Center/National Clinical Research Center for Cancer/Cancer Hospital, Chinese Academy of Medical Sciences and Peking Union Medical College, Beijing, China

**Keywords:** HER2-low, HER2-zero, breast cancer, prognosis, meta-analysis

## Abstract

**Background:**

HER2-low expression breast cancer (BC) accounts for approximately 45%-55% of all BC cases. The purpose of this study was to investigate the prognostic difference between patients with HER2-low expression and HER2-zero BC.

**Methods:**

An electronic search of Pubmed, Embase, Cochrane Library, and Web of Science databases was performed to screen studies that included prognostic comparisons between HER2-zero and HER2-low expression groups. A total of 14 studies involving 52106 patients were included.

**Results:**

Our results indicated that HER2-low expression was associated with a significant benefit in OS among all patients with early BC (HR, 0.83; 95% CI, 0.78–0.88), patients with hormone-receptor positive BC (HR, 0.83; 95% CI, 0.77–0.89), and patients with TNBC (HR, 0.78; 95% CI, 0.70–0.87). HER2-low expression was associated with a significant benefit in DFS among all patients (HR, 0.81; 95% CI, 0.71–0.93), patients with hormone receptor-positive BC (HR, 0.81; 95% CI, 0.72–0.90), but no significant difference in DFS was found in patients with TNBC (HR, 0.87; 95% CI, 0.65–1.17). HER2-low expression was associated with a significant benefit in RFS among all patients (HR, 0.90; 95% CI, 0.85–0.95), patients with hormone receptor-positive BC (HR, 0.90; 95% CI, 0.84–0.96), but no significant difference in RFS was found in patients with TNBC (HR, 0.80; 95% CI, 0.55–1.16).

**Conclusions:**

Among patients with early-stage BC, patients with HER2-low expression BC had better OS in the overall population, hormone receptor-positive and TNBC subgroups. Besides, favorable DFS and RFS were observed in both the overall population and hormone receptor-positive subgroup.

**Systematic review registration:**

https://www.crd.york.ac.uk/PROSPERO/, identifier (CRD 42022349458).

## 1 Introduction

Breast cancer (BC) is the most commonly diagnosed cancer among women worldwide. According to the Global Cancer Statistics 2020, there were an estimated 2.3 million new cases of female BC worldwide in 2020 (1). Human epidermal growth factor receptor 2 (HER2) detection is essential for BC treatment planning. HER2-positive BC accounts for approximately 15% of all BC cases, in which multiple agents targeting HER2 have provided significant clinical benefits in patients with both early and advanced BC (2, 3). However, 85% of patients with BC were classified as HER2-negative and were therefore ineligible for anti-HER2 treatment (4). Recently, antibody-drug conjugates (ADCs) have been proved to have antitumor activity in patients with classical HER2-positive BC (5, 6), as well as BC with low HER2 expression (7). These results have led to the concept of “HER2-low expression” which includes tumors with HER2 expression indicated by a immunohistochemistry (IHC) score of 1+ or 2+/fluorescence *in-situ* hybridization (FISH)-negative.

In the past, HER2-low expression and HER2-zero BC have been combined and considered HER2 negative BC. Moreover, HER2-low expression BC accounts for approximately 45%–55% of all BC cases, indicating that the number of new HER2 low-expression BC cases could be approximately 1 million worldwide annually, which is almost equivalent to that of all new annual gastric cancer cases worldwide (1, 4). Because the population of patients with BC with HER2-low expression is very large, understanding the associations of different clinicopathologic characteristics and prognosis between patients with HER2-low expression and HER2-zero BC is significant, and will help clinicians develop more precise treatment strategies and avoid overtreatment or undertreatment in patients with HER2-low expression BC in the future. In addition, it may guide the design of future clinical trials for HER2-low expression BC.

Several studies have shown that compared with HER2-zero BC, HER2-low expression BC has a specific biology with varying responses to therapy and prognosis (8–10). However, other studies have found that HER2-low expression is indistinct from HER2-zero BC in terms of clinicopathologic characteristics and prognosis (11). Thus, whether HER2-low expression BC varies in biological and prognostic significance from that of HER2-zero BC remains unclear. This study aimed to evaluate the biological and prognostic significance of HER2-low expression in patients with BC.

## 2 Method

The study protocol adhered to the Preferred Reporting Items for Systematic Reviews and Meta-analyses (PRISMA) reporting guidelines (12, 13). This systematic review was prospectively registered with The International Prospective Register of Systematic Reviews (CRD 42022349458). Because this study was based exclusively on published literature, ethics approval and informed consent were not required.

### 2.1 Study objectives

The primary objective was to identify associations between prognosis, including overall survival (OS), disease-free survival (DFS), and relapse-free survival (RFS), and early HER2-low expression (HER2 IHC 1/2+ with FISH negative) and HER2-zero BC, including hormone receptor-positive BC and triple-negative BC (TNBC). The secondary objective was to identify associations between prognosis, including OS and RFS, and early HER2 IHC 0, HER2 IHC 1+, and HER2 IHC 2+ (IHC 2+ in the following text refers to IHC 2+/FISH-negative) BC, including hormone receptor-positive BC and TNBC. In addition, subgroup analyses were performed. The association of DFS and distant DFS (DDFS) with HER2-low in high-genetic-risk and low-genetic-risk groups was analyzed.

### 2.2 Literature search

We conducted an electronic search of PubMed, Embase, Cochrane Library, and Web of Science databases. The search strategy combined Medical Subject Heading terms and keywords encompassing two key concepts: BC and HER2-low expression (**Supplementary Table 1**). All titles were initially screened independently and the appropriate abstracts were reviewed independently by two authors (T.W. and DY.W.). Abstracts that met the criteria were retained for full-text review. Disagreements were resolved through discussion during the screening and extraction period.

### 2.3 Study selection

The selected studies had to meet the following inclusion criteria simultaneously: (1) published from January 1, 2015, to July 21, 2022 in English; (2) study population included patients with early BC; (3) analysis included prognostic comparisons between HER2-zero and HER2-low expression groups or between any two groups among HER2-zero, HER2 IHC 1+, and HER2 IHC 2+ groups (e.g., HER2-zero and HER2 IHC 1+ vs. HER2 IHC 2+); (4) OS, DFS or RFS were reported as hazard ratios (HRs) (If no HRs were presented for OS, DFS, or RFS, the Kaplan-Meier [K-M] curve of any OS, DFS, or RFS outcome must be provided to facilitate data extraction of K-M curves to calculate HRs); (5) retrospective study, randomized controlled trial (RCT), or pool analysis study. Regarding studies with populations comprised of patients with both early and advanced or metastatic BC, the prognostic analysis must have been performed separately for patients with early BC; otherwise, the proportion of patients with advanced or metastatic BC must be less than 10%.

The exclusion criteria were studies (1) published in a language other than English or before January 1, 2015 (2) in which populations included mainly advanced or metastatic BC without separate prognostic analysis of patients with early BC or (3) without survival comparisons of OS, DFS, or RFS between patients with HER2-zero and HER2-low expression BC,or among HER2-zero, HER2 IHC 1+, and HER2 IHC 2+ groups.

### 2.4 Data extraction

Study and participant characteristics and outcome measures were extracted by two authors (T.W. and DY.W.) independently. Disagreements were resolved by discussion until consensus. The following variables were extracted: title and study details (year, journal, and location), study population characteristics (sample size, median age, median follow-up, tumor size, lymph node status, tumor grade, stage), and outcome data. The HRs for OS, DFS, and RFS were extracted from each eligible study. If K-M curves were provided without HRs in the reported literature, we used Engauge Digitizer to extract data from K-M curves and calculate the respective HRs using the practical methods described by Tierney et al. (14).

### 2.5 Statistical analysis

Statistical analysis was performed from August 15, 2022, to August 25, 2022. Outcome data were reported as HRs; If K-M curves were provided without HRs, HRs were calculated using data extracted from K-M curves. The 95% confidence intervals (CIs) were estimated using the Mantel-Haenszel method. The 95% CIs that did not cross unity were considered statistically significant. *I^2^* statistics were used to estimate statistical heterogeneity, with greater than 50% indicating significant heterogeneity. When no significant heterogeneity (*I^2^* ≤ 50%) was observed, a fixed-effects model was used. In contrast, when significant heterogeneity (*I^2^* > 50%) was observed, a random-effects model was used to calculate the pooled effect estimate (HR) to explain any possible inter-study heterogeneity. Sensitivity analysis was performed to assess the robustness of the meta-analysis conclusions. Two-sided and *P* values <0.05 were considered statistically significant in all analyses. All statistical analyses were performed using R, version 4.1.1 (R Foundation for Statistical Computing, Vienna, Austria).

### 2.6 Risk of bias

Risk of bias (RoB) was assessed by two authors (T.W. and DY.W.). Retrospective studies were assessed using the Newcastle-Ottawa Scale based on several parameters, including patient selection, ascertainment of exposure, outcome assessment, cohort comparability, and follow-up duration and adequacy (15). Points were calculated for each study and classified as low, high, or unclear RoB accordingly. Disagreements regarding these categories were resolved through discussion until consensus between the authors was reached. The Egger test was used for funnel plot asymmetry and to visualize publication bias (16).

## 3 Results

### 3.1 Characteristics of the included studies

**Figure 1:**
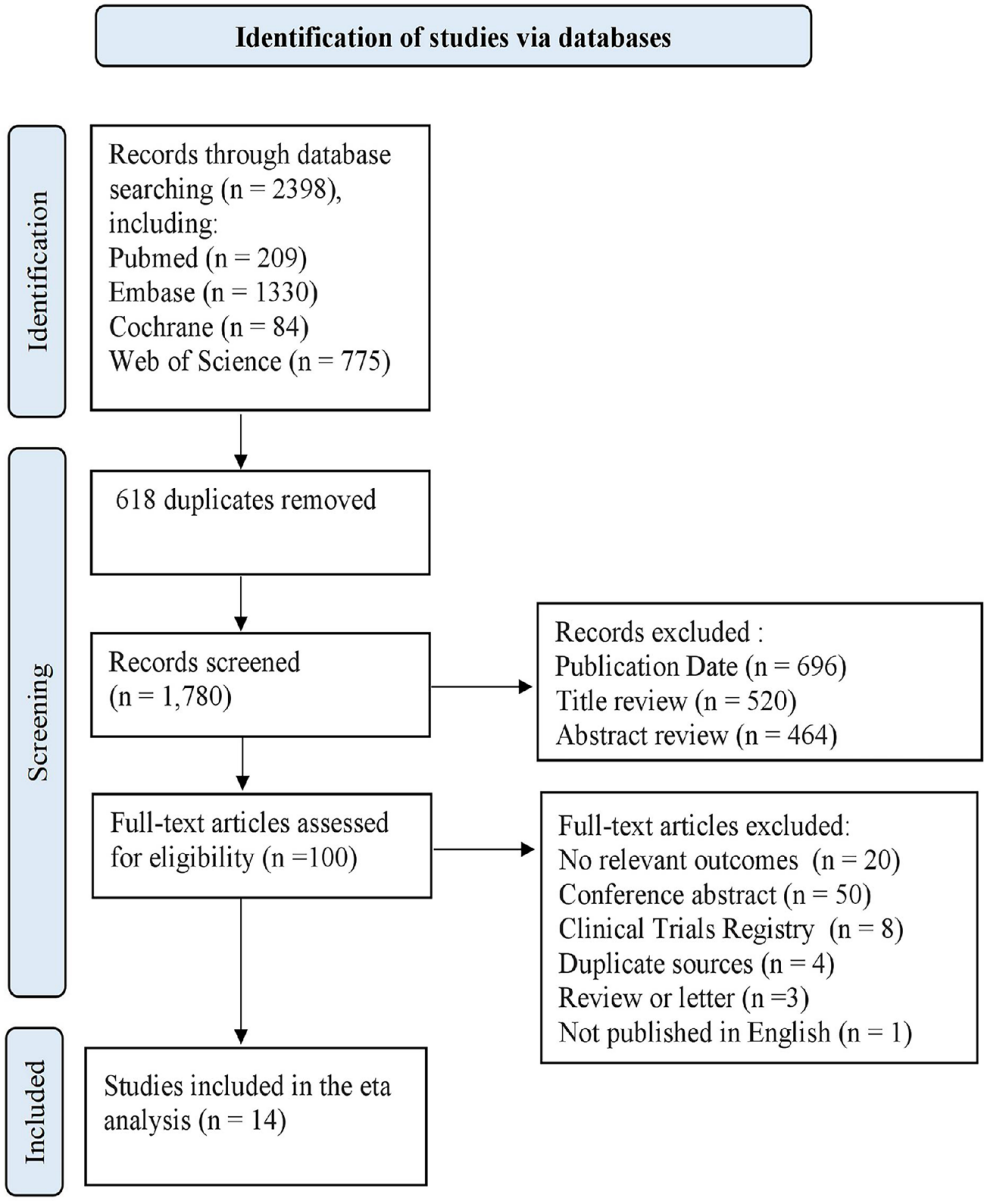
Flow diagram of search strategy and study selection.

The flow diagram (**Figure 1**) outlines the study selection process and reasons for exclusion. In total, 2398 publications were identified using the predefined search terms, of which 14 studies met the inclusion criteria (17–30).

Among the 14 selected articles, 52106 participants were ultimately included in the analysis. One pool analysis (17) and one RCT (30) were included, whereas the remaining 12 studies were retrospective cohort studies. For 3 studies that did not include K-M curves with HRs, the HRs were estimated using data extracted from the K-M curves. The two reviewers were in 100% agreement regarding the extracted data. **Supplementary Table 2** provides an overview of the main characteristics and relevant outcomes of the included studies. Included studies were assessed according to Newcastle-Ottawa scores, which are summarized in **Supplementary Table 3**. None of the included studies was classified as having a high RoB for objective outcomes. The included studies differed in their methodology. The periods ranged from 0.8 to 10.3 years. The sample sizes ranged from 296 to 5235 patients. Moreover, 4 studies were conducted in Europe, 6 studies in Asia, 1 study in North America, 1 study in South America, and 2 studies in Multi-continents. The mean age of the patients varied from 45.3 to 66.1 years old.

### 3.2 OS

**Figure 2:**
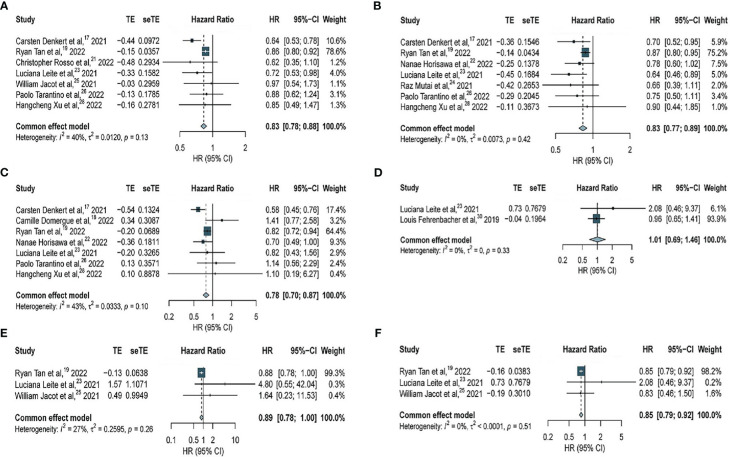
Forest plot of **(A)** OS in overall EBC population (HER2 low vs. HER2 0); **(B)** OS in hormone receptor positive subgroup (HER2 low vs. HER2 0); **(C)** OS in TNBC subgroup (HER2 low vs. HER2 0); **(D)** OS in overall EBC population (HER2 IHC 2 vs. IHC 1); **(E)** OS in overall EBC population (HER2 IHC 2 vs. IHC 0); **(F)** OS in overall EBC population (HER2 IHC 1 vs. IHC 0).

In this meta-analysis, 7 studies with 37466 patients were included to assess the association of HER2-low expression and HER2-zero BC with OS among all patients (including patients with hormone receptor-positive BC and TNBC) with early BC. Our results indicated that among all patients with early BC, HER2-low expression was associated with a significant benefit in OS (HR, 0.83; 95% CI, 0.78–0.88), with low heterogeneity observed across studies (*I^2^* = 40%; *P* = 0.13) (**Figure 2A**).

Furthermore, 7 studies with 34229 patients and 7 studies with 7482 patients were included to assess the association of HER2-low expression and HER2-zero BC with OS in patients with hormone receptor-positive BC and TNBC, respectively. HER2-low expression was significantly associated with longer OS in patients with hormone-receptor positive BC (HR, 0.83; 95% CI, 0.77–0.89), with low heterogeneity observed across studies (*I^2^* = 0%; *P* = 0.42) (**Figure 2B**). Similarly, in patients with TNBC, HER2-low expression was significantly associated with longer OS (HR, 0.78; 95% CI, 0.70–0.87), with moderate heterogeneity observed across studies (*I^2^* = 43%; *P* = 0.10) (**Figure 2C**).

To determine whether HER2-zero, HER2 IHC 1+, and HER2 IHC 2+ BC were associated with OS among all patients (including patients with hormone receptor-positive BC and TNBC), further analyses were performed. Two studies with 3490 patients revealed no significant difference in OS between BC patients with HER2 IHC 2+ and HER2 IHC 1+ (HR, 1.01; 95% CI, 0.69–1.46), with no considerable heterogeneity (*I^2^* = 0%; *P* = 0.33) (**Figure 2D**). However, data obtained from three studies with 20407 patients revealed no significant difference in OS between patients with HER2 IHC 2+ and HER2-zero BC (HR, 0.89; 95% CI, 0.78–1.00),with no considerable heterogeneity (*I^2^* = 27%; *P* = 0.26) (**Figure 2E**). Significantly longer OS was observed in patients with HER2 IHC 1+ than that in HER2-zero based on three studies with 25910 patients (HR, 0.85; 95% CI, 0.79–0.92), with no considerable heterogeneity (*I^2^* = 0%; *P* = 0.51) (**Figure 2F**).

### 3.3 DFS

**Figure 3:**
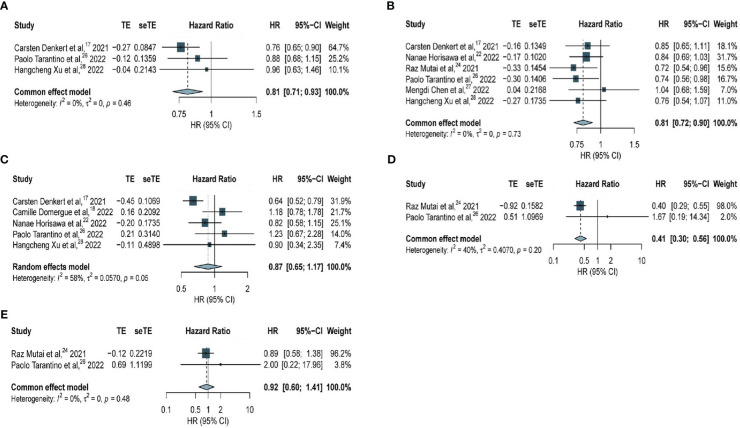
Forest plot of **(A)** DFS in overall EBC population (HER2 low vs. HER2 0); **(B)** DFS in hormone receptor positive subgroup (HER2 low vs. HER2 0); **(C)** DFS in TNBC subgroup (HER2 low vs. HER2 0); **(D)** DFS in high genetic risk EBC population (HER2 low vs. HER2 0); **(E)** DFS in low genetic risk EBC population (HER2 low vs. HER2 0).

Among all patients (including patients with hormone receptor-positive BC and TNBC), significantly longer DFS was observed in patients with HER2-low expression compared with that in patients with HER2-zero BC (HR, 0.81; 95% CI, 0.71–0.93) based on three studies with 7667 patients, with no considerable heterogeneity observed (*I^2^* = 0%; *P* = 0.46) (**Figure 3A**).

Regarding patients with hormone receptor-positive BC, the analysis based on six studies with 12283 patients revealed significantly longer DFS among patients with HER2-low expression compared with that in patients with HER2-zero BC (HR, 0.81; 95% CI, 0.72–0.90), with no considerable heterogeneity observed (*I^2 =^* 0%; *P* = 0.73) (**Figure 3B**). The association of HER2-low expression and DDFS in hormone receptor-positive BC was analyzed (**Supplementary Figure 1A**) and no significant difference was observed based on two studies with 5146 patients (HR, 0.73; 95% CI, 0.59–0.91), with no considerable heterogeneity observed (*I^2^* = 0%; *P* = 0.79).

However, among patients with TNBC, no significant difference in DFS was found in patients with HER2-low expression and HER2-zero BC (HR, 0.87; 95% CI, 0.65–1.17) based on five studies with 2535 patients, and this outcome was statistically insignificant within a very heterogeneous study group (*I^2^* = 58%, *P* = 0.05) (**Figure 3C**).

Further analyses were performed to determine whether genetic risk was associated with DFS among all patients. Among all patients with high genetic risk, significantly longer DFS was observed among patients with HER2-low expression compared with that in patients with HER2-zero BC (HR, 0.41; 95% CI, 0.30–0.56) based on data obtained from two studies with 392 patients, with no considerable heterogeneity observed (*I^2^* = 40%; *P* = 0.20) (**Figure 3D**). Among all patients with low genetic risk, data obtained from two studies with 1956 patients revealed no significant difference in DFS between patients with HER2-low expression and HER2-zero BC (HR, 0.92; 95% CI, 0.60–1.41), with no considerable heterogeneity observed (*I^2 =^* 0%; *P* = 0.48) (**Figure 3E**).

The same association was observed for DDFS. No significant difference was observed in patients with low genetic risk based on two studies with 1956 patients (**Supplementary Figure 1B**), whereas patients with HER2-low expression had significantly better DDFS compared with that in patients with high genetic risk based on two studies with 392 patients (**Supplementary Figure 1C**).

### 3.4 RFS

**Figure 4:**
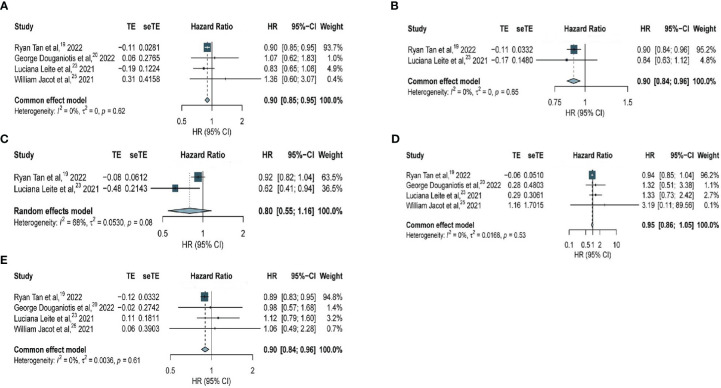
Forest plot of **(A)** RFS in overall EBC population (HER2 low vs. HER2 0); **(B)** RFS in hormone receptor positive subgroup (HER2 low vs. HER2 0); **(C)** RFS in TNBC subgroup (HER2 low vs. HER2 0); **(D)** RFS in overall EBC population (HER2 IHC 2 vs. IHC 0); **(E)** RFS in overall EBC population (HER2 IHC 1 vs. IHC 0).

Among all patients, patients with HER2-low expression had significantly longer RFS compared with that in patients with HER2-zero BC (HR, 0.90; 95% CI, 0.85–0.95) based on four studies with 30380 patients, with no considerable heterogeneity observed (*I^2^* = 0%; *P* = 0.62) (**Figure 4A**). Regarding patients with hormone receptor-positive BC, our analysis of two studies with 24045 patients revealed significantly longer RFS among patients with HER2-low expression compared with that in patients with HER2-zero BC (HR, 0.90; 95% CI, 0.84–0.96), with no considerable heterogeneity observed (*I^2^* = 0%; *P* = 0.65) (**Figure 4B**). However, among patients with TNBC, no significant difference was seen in patients with HER2-low expression and HER2-zero BC (HR, 0.80; 95% CI, 0.55–1.16) based on two studies with 4947 patients and within a very heterogeneous study group (*I^2^* = 68%, *P* = 0.053) (**Figure 4C**).

An analysis of the association of HER2-zero, HER2 IHC 1+, and HER2 IHC 2+ BC with RFS was performed. Among all patients, data obtained from four studies with 20884 patients revealed no significant difference in RFS between patients with HER2 IHC 2+ and HER2-zero BC (HR, 0.95; 95% CI, 0.86–1.05), with no considerable heterogeneity observed (*I^2^* = 0%; *P* = 0.53) (**Figure 4D**). However, significantly longer RFS was observed in patients with HER2 IHC 1+ than that in patients with HER2-zero BC (HR, 0.90; 95% CI, 0.84–0.96) based on four studies with 26699 patients, with no considerable heterogeneity observed (*I^2^* = 0%; *P* = 0.61) (**Figure 4E**).

It’s worth mentioning that sensitivity analyses were performed for each of these analyses (**Supplementary Figures 2**-**4**). The sensitivity analysis suggested that the study by Denkert et al. (17) was the source of heterogeneity in the analysis of TNBC. Denkert et al. (17) is a pool analysis of four RCTs, and different study design types may be the source of heterogeneity. Therefore, the analysis was performed again after removing the study by Denkert et al. (17), and the results showed that HER2-low expression was still significantly associated with longer OS (HR, 0.83; 95% CI, 0.74–0.94), with low heterogeneity observed across studies (*I^2^* = 0%; *P* = 0.45), consistent with our previous results (**Supplementary Figure 5A**). And HER2-low expression was still significantly associated with longer DFS (HR, 0.99; 95% CI, 0.77–1.28), with low heterogeneity observed across studies (*I^2 =^* 0%; *P* = 0.49) which is consistent with the results of the analysis of keeping the study by Denkert et al. (17) (**Supplementary Figure 5B**). Besides, Egger test was used for funnel plot asymmetry and no significant publication bias was found except the analysis for OS in patients with hormone-receptor positive BC (**Supplementary Figure 6**).

## 4 Discussion

Recently, the remarkable therapeutic effect of novel ADCs on HER2-low expression BC has generated great interest for this new subtype. Nevertheless, the prognosis of HER2-low expression BC remains controversial. In our systematic review and meta-analysis of the published data, the prognostic difference between patients with HER2-low expression and HER2-zero BC was analyzed among patients with early-stage BC, both in the overall population and hormone receptor-positive and TNBC subgroups.

We found that compared with patients with HER2-zero BC, patients with HER2-low expression BC had better OS, DFS, and RFS both in the overall population and hormone receptor-positive subgroup, suggesting distinct biological subtype of HER2-low expression. In the TNBC subgroup, OS was superior in patients with HER2-low expression compared with that for patients with HER2-zero BC, whereas no significant differences in DFS and RFS were observed between patients with HER2-low expression and HER2-zero BC.

Among all patients with BC, significantly longer OS and RFS was observed in patients with HER2 IHC 1+ compared with that in patients with HER2-zero BC. However, no significant difference in OS and RFS was observed between patients with HER2 IHC 2+ and HER2-zero BC. No significant difference in OS was observed between patients with HER2 IHC 1+ and HER2-zero BC. The prognostic difference in RFS between patients with HER2 IHC 1+ and HER2-zero BC was not analyzed because of data leakage.

In addition, the Oncotype Dx risk score was compared between HER2-low expression and HER2-zero BC among patients with hormone receptor-positive BC (24, 26). Interestingly, the prognosis of HER2-low expression BC differs significantly in patients with high genetic risk (Oncotype Dx risk score > 26), although not for patients with low genetic risk (Oncotype Dx risk score ≤ 25). In early hormone receptor-positive BC with high genomic risk, HER2-low expression was associated with more favorable DFS and DDFS compared with that for HER2-zero BC. However, for early hormone receptor-positive BC with low genomic risk, no significant differences were observed in DFS or DDFS between patients with HER2-low expression and HER2-zero BC.

The findings of a recent study involving 30491 patients support that HER2-low expression has a better prognosis than that of HER2-zero BC, although this study used BC-specific survival as an outcome indicator, which was not included in our meta-analysis. This conclusion is consistent with our findings and further validates our conclusion (8).

Several reasons may explain why HER2-low expression has a more favorable prognosis in patients with hormone receptor-positive BC, whereas only OS was superior in patients with TNBC. First, the PAM50 intrinsic subtype profiles of HER2-low expression BC were demonstrated in a recent study (9), which concluded that in hormone receptor-positive BC, the gene expression of patients with HER2-zero and HER2-low expression tumors was obviously different. However, no significant difference in gene expression was observed between HER2-zero and HER2-low expression in patients with TNBC. This indicates that HER2-low expression is more likely to be a distinct biological entity in hormone receptor-positive than in TNBC tumors.

What’s more, several studies have reported an association between hormone receptor status and HER2-low expression. The percentage of HER2-low expression differed between the hormone receptor-positive and TNBC groups. Interestingly, the prevalence of HER2-low expression was higher in patients with hormone receptor-positive BC than that in TNBC (9, 31). HER2-low expression BC tends to be luminal-like with high estrogen receptor (ER) expression, whereas HER2-zero BC is generally more basal-like, with low ER expression (26). ER expression may play a confounding role when analyzing the prognostic difference between patients with HER2-low expression and HER2-zero BC in some studies. However, this hypothesis requires further statistical analysis and verification in future studies.

Further, HER2-low expression BC is reportedly associated with indicators of decreased aggressiveness, such as lower histological grade, lower Ki-67 status, and fewer TP53 mutations compared with that of HER2-zero BC (17). Whether the prognostic differences are driven by HER2-low expression or the varying distribution of other clinicopathological characteristics, such as ER expression, requires further investigation.

HER2-low expression and HER2-zero BC vary in the somatic mutation landscape. In patients with HER2-low expression BC, the frequency of phosphatidylinositol 3-kinase/protein kinase B signaling mutations was higher, and the frequency of p53 signaling and cell cycle pathway mutations was lower. This conclusion supports that HER2-low expression and HER2-zero BC are two different entities (10). Another study with similar findings reported that PIK3CA and TP53 mutation frequencies differed between patients with HER2-low expression and HER2-zero BC. Moreover, BRCA1/2 and other BC predisposition gene mutations have different frequencies (17). Studies on the PAM50 intrinsic subtype also found significant differences in gene expression between HER2-low expression and HER2-zero among patients with hormone receptor-positive BC, although no significant differences were observed in patients with TNBC (9). Further studies are needed to verify whether different gene expression profiles lead to different prognoses, and whether these differences are sufficient for classification into independent molecular subtypes.

Owing to the promising future of ADCs in treating HER2-low expression BC, researchers are conducting clinical trials to investigate the therapeutic effect of advanced treatment with novel ADCs in patients with early stage BC. However, we observed significant survival differences between patients with HER2-low expression and HER2-zero BC. This study suggests the possibility that patients with HER2-low expression BC may receive de-escalated treatment to achieve the desired therapeutic effect, which could guide the design of future clinical trials. Our results provide new directions for future research.

Many studies have found poor concordance between different pathologists when using IHC to assess HER2-low expression and HER2-zero BC. One study found that there was only 26% agreement when IHC was used to assess low levels of HER2 (i.e., IHC 0 and IHC 1+) (32). The phase 1b trastuzumab deruxtecan study reported consistency of 40% for HER2 IHC 2+ and 70% for HER2 IHC 1+ between local and central pathology reports (33). This suggests that pathologists need to use more accurate methods to distinguish HER2-low expression from HER2-zero in the future, such as the detection of mRNA expression or quantitative automated chemistry.

## 5 Limitations

Several limitations of this study should be considered when interpreting the results. First of all, considering the accessibility and quality of the available literature, only studies published in English were included in our study. Considering the integrity of the data, conference reports were not included in the report, which potentially affected the interpretation of our results. Secondly, to fully utilize the data, if K-M curves were provided without HRs, we used Engauge Digitizer to extract data from K-M curves and calculated the HRs using practical methods and a small data set. Owing to inevitable human errors when using measurement tools, a certain degree of deviation might exist between the extracted and real HRs. Thirdly, this meta-analysis included retrospective studies, RCT, and pool analysis, which may have increased the heterogeneity among studies. And the only RCT (30) was treated as a cohort study and the RCT was assessed using the Newcastle-Ottawa Scale. In the analysis of OS and DFS among patients with TNBC, the heterogeneity was large (with *I^2^* values of 43% and 58%, respectively). To reduce the impact of this possible heterogeneity on the results, the sensitivity analysis was performed. After the sensitivity analysis, we concluded that the main source of heterogeneity was from the included pool analysis study (17). Therefore, we excluded this article and conducted another meta-analysis of patients with TNBC with OS and DFS as outcome indicators. Nevertheless, we reached similar conclusions. In addition, there were relatively small number of studies for each analysis which limited further analysis for whether the length of follow-up duration or the different therapy method have influence on the conclusion. Further studies are needed. Lastly, in the analysis of the prognostic differences among genetic risk types and HER2 IHC groups, the number of included studies was small. The sensitivity analysis was conducted to evaluate the robustness of the meta-analysis results.

Despite these limitations, we believe that this analysis provides significant implications for future treatment strategies and research directions.

## 6 Conclusion

Whether HER2 low is a prognostic factor in early BC is widely discussed and has attracted the attention of many scholars. Nevertheless, the prognosis of HER2-low expression BC is still controversial at present. Therefore, the study aimed to evaluate the prognostic significance of HER2-low expression in patients with BC. Overall, this meta-analysis revealed that among patients with early-stage BC, patients with HER2-low expression BC had better OS in the overall population and hormone receptor-positive and TNBC subgroups. In particular, favorable DFS and RFS were observed in both the overall population and hormone receptor-positive subgroup. The results of this meta-analysis support that there are distinct subtypes of HER2-low expression BC, although further studies are necessary to verify whether differences in genetic profiles are sufficient for classification into independent molecular subtypes. The results of this meta-analysis will deepen the general understanding of HER2-low expression BC and have important implications for future therapeutic strategies.

## Data availability statement

The original contributions presented in the study are included in the article/**Supplementary Material**. Further inquiries can be directed to the corresponding author.

## Author contributions

TW, DW, SG, and PY contributed to the conception of the study, performed the data analyses and wrote the manuscript. XW, JY, YK, JJ, ZY, and YS helped perform the analysis with constructive discussions. All authors contributed to the article and approved the submitted version.

## Supplementary material

Supplementary Figure 1 Forest plot of **(A)** DDFS in hormone receptor positive subgroup (HER2 low vs. HER2 0) **(B)** DDFS in low genetic risk EBC population (HER2 low vs. HER2 0) **(C)** DDFS in high genetic risk EBC population (HER2 low vs. HER2 0)

Supplementary Figure 2 Sensitivity analysis of **(A)** OS in overall EBC population (HER2 low vs. HER2 0); **(B)** OS in hormone receptor positive subgroup (HER2 low vs. HER2 0); **(C)** OS in TNBC subgroup (HER2 low vs. HER2 0); **(D)** OS in overall EBC population (HER2 IHC 2 vs. IHC 0); **(E)** OS in overall EBC population (HER2 IHC 1 vs. IHC 0).

Supplementary Figure 3 Sensitivity analysis of **(A)** DFS in overall EBC population (HER2 low vs. HER2 0); **(B)** DFS in hormone receptor positive subgroup (HER2 low vs. HER2 0); **(C)** DFS in TNBC subgroup (HER2 low vs. HER2 0).

Supplementary Figure 4 Sensitivity analysis of **(A)** RFS in overall EBC population (HER2 low vs. HER2 0); **(B)** RFS in overall EBC population (HER2 IHC 2 vs. IHC 0); **(C)** RFS in overall EBC population (HER2 IHC 1 vs. IHC 0).

Supplementary Figure 5 Forest plot of **(A)** OS in TNBC subgroup (HER2 low vs. HER2 0) removed an article with potential heterogeneity; **(B)** DFS in TNBC subgroup (HER2 low vs. HER2 0) removed an article with potential heterogeneity;

Supplementary Figure 6 Egger test of **(A)** OS in overall EBC population (HER2 low vs. HER2 0), p=0.3675; **(B)** OS in hormone receptor positive subgroup (HER2 low vs. HER2 0), p=0.0435; **(C)** OS in TNBC subgroup (HER2 low vs. HER2 0), p=0.5758; **(D)** DFS in hormone receptor positive subgroup (HER2 low vs. HER2 0), p=0.7471; **(E)** DFS in TNBC subgroup (HER2 low vs. HER2 0), p=0.1468.
