# Supplementary material for: Clinicopathologic characteristics and prognostic significance of HER2-low expression in patients with early breast cancer: A systematic review and meta-analysis
Source: Front Oncol. 2023 Feb 2;13:1100332. doi: 10.3389/fonc.2023.1100332 (PMC9931719; doi:10.3389/fonc.2023.1100332)
Supplement: Supplementary Figure 1 — Forest plot of (A) DDFS in hormone receptor positive subgroup (HER2 low vs. HER2 0) (B) DDFS in low genetic risk EBC population (HER2 low vs. HER2 0) (C) DDFS in high genetic risk EBC population (HER2 low vs. HER2 0) [file DataSheet_1.zip › Supplementary Tables and Figures/Supplementary Table 1. Detailed Search Strategy.docx]

Supplementary Table 1. Detailed Search Strategy

| Database | PICOS | Search strategy |
| --- | --- | --- |
| Pubmed | P | **("Breast Neoplasms"[Mesh] OR** "Breast Neoplasm"[Title] OR "Neoplasm, Breast"[Title] OR "Breast Tumors"[Title] OR "Breast Tumor"[Title] OR "Tumor, Breast"[Title] OR "Tumors, Breast"[Title] OR "Neoplasms, Breast"[Title] OR "Breast Cancer"[Title] OR "Cancer, Breast"[Title] OR "Mammary Cancer"[Title] OR "Cancer, Mammary"[Title] OR "Cancers, Mammary"[Title] OR "Mammary Cancers"[Title] OR "Malignant Neoplasm of Breast"[Title] OR "Breast Malignant Neoplasm"[Title] OR "Breast Malignant Neoplasms"[Title] OR "Malignant Tumor of Breast"[Title] OR "Breast Malignant Tumor"[Title] OR "Breast Malignant Tumors"[Title] OR "Cancer of Breast"[Title] OR "Cancer of the Breast"[Title] OR "Mammary Carcinoma, Human"[Title] OR "Carcinoma, Human Mammary"[Title] OR "Carcinomas, Human Mammary"[Title] OR "Human Mammary Carcinomas"[Title] OR "Mammary Carcinomas, Human"[Title] OR "Human Mammary Carcinoma"[Title] OR "Mammary Neoplasms, Human"[Title] OR "Human Mammary Neoplasm"[Title] OR "Human Mammary Neoplasms"[Title] OR "Neoplasm, Human Mammary"[Title] OR "Neoplasms, Human Mammary"[Title] OR "Mammary Neoplasm, Human"[Title] OR "Breast Carcinoma"[Title] OR "Breast Carcinomas"[Title] OR "Carcinoma, Breast"[Title] OR "Carcinomas, Breast"[Title]) AND (“HER2 Low”[Title/Abstract] OR “Low HER2”[Title/Abstract] OR “Low Level HER2 Expression”[Title/Abstract] OR “HER2 Zero”[Title/Abstract]) |
| Embase | P | ('breast cancer'/exp OR 'ca breast' OR 'breast cancer' OR 'breast gland cancer' OR 'breast gland neoplasm' OR 'breast malignancies' OR 'breast malignancy' OR 'breast tumor malignant' OR 'cancer in the mammary gland' OR 'cancer of the breast OR 'cancer of the mammary gland' OR 'cancer, breast' OR 'malignancies of the breast' OR 'malignancy of the breast' OR 'malignant breast neoplasm' OR 'malignant breast tumor' OR 'malignant neoplasm of the breast' OR 'malignant tumor of the breast' OR 'mamma cancer OR 'mammary cancer' OR 'mammary gland cancer' OR 'mammary gland malignancy' OR 'mammary malignancies' OR 'mammary malignancy') AND [(low NEAR/4 her2):ab,ti] |
| Cochrane Library | P | {(MeSH descriptor: [Breast Neoplasms] explode all trees) OR [("Breast Neoplasm"):ti OR ("Neoplasm, Breast"):ti OR ("Breast Tumors"):ti OR ("Breast Tumor"):ti OR ("Tumor, Breast"):ti OR ("Tumors, Breast"):ti OR ("Neoplasms, Breast"):ti OR ("Breast Cancer"):ti OR ("Cancer, Breast"):ti OR ("Mammary Cancer"):ti OR ("Cancer, Mammary"):ti OR ("Cancers, Mammary"):ti OR ("Mammary Cancers"):ti OR ("Malignant Neoplasm of Breast"):ti OR ("Breast Malignant Neoplasm"):ti OR ("Breast Malignant Neoplasms"):ti OR ("Malignant Tumor of Breast"):ti OR ("Breast Malignant Tumor"):ti OR ("Breast Malignant Tumors"):ti OR ("Cancer of Breast"):ti OR ("Cancer of the Breast"):ti OR ("Mammary Carcinoma, Human"):ti OR ("Carcinoma, Human Mammary"):ti OR ("Carcinomas, Human Mammary"):ti OR ("Human Mammary Carcinomas"):ti OR ("Mammary Carcinomas, Human"):ti OR ("Human Mammary Carcinoma"):ti OR ("Mammary Neoplasms, Human"):ti OR ("Human Mammary Neoplasm"):ti OR ("Human Mammary Neoplasms"):ti OR ("Neoplasm, Human Mammary"):ti OR ("Neoplasms, Human Mammary"):ti OR ("Mammary Neoplasm, Human"):ti OR ("Breast Carcinoma"):ti OR ("Breast Carcinomas"):ti OR ("Carcinoma, Breast"):ti OR ("Carcinomas, Breast"):ti ]} AND [(low NEAR/4 her2):ab] |
| Web of Science | P | **[((((((((((((((((((((((((((((((((((((TI=("Breast Neoplasm")) OR TI=("Neoplasm, Breast")) OR TI=("Breast Tumors")) OR TI=("Breast Tumor")) OR TI=("Tumor, Breast")) OR TI=("Tumors, Breast")) OR TI=("Neoplasms, Breast")) OR TI=("Breast Cancer")) OR TI=("Cancer, Breast")) OR TI=("Mammary Cancer")) OR TI=("Cancer, Mammary")) OR TI=("Cancers, Mammary")) OR TI=("Mammary Cancers")) OR TI=("Malignant Neoplasm of Breast")) OR TI=("Breast Malignant Neoplasm")) OR TI=("Breast Malignant Neoplasms")) OR TI=("Malignant Tumor of Breast")) OR TI=("Breast Malignant Tumor")) OR TI=("Breast Malignant Tumors")) OR TI=("Cancer of Breast")) OR TI=("Cancer of the Breast")) OR TI=("Mammary Carcinoma, Human")) OR TI=("Carcinoma, Human Mammary")) OR TI=("Carcinomas, Human Mammary")) OR TI=("Human Mammary Carcinomas")) OR TI=("Mammary Carcinomas, Human")) OR TI=("Human Mammary Carcinoma")) OR TI=("Mammary Neoplasms, Human")) OR TI=("Human Mammary Neoplasm")) OR TI=("Human Mammary Neoplasms")) OR TI=("Neoplasm, Human Mammary")) OR TI=("Neoplasms, Human Mammary")) OR TI=("Mammary Neoplasm, Human")) OR TI=("Breast Carcinoma")) OR TI=("Breast Carcinomas")) OR TI=("Carcinoma, Breast")) OR TI=("Carcinomas, Breast")]AND [**AB=(low near/4 HER2)**]** |

PICOS: population, intervention, comparison, outcomes and study design;
