# Supplementary material for: Clinicopathologic characteristics and prognostic significance of HER2-low expression in patients with early breast cancer: A systematic review and meta-analysis
Source: Front Oncol. 2023 Feb 2;13:1100332. doi: 10.3389/fonc.2023.1100332 (PMC9931719; doi:10.3389/fonc.2023.1100332)
Supplement: Supplementary Figure 1 — Forest plot of (A) DDFS in hormone receptor positive subgroup (HER2 low vs. HER2 0) (B) DDFS in low genetic risk EBC population (HER2 low vs. HER2 0) (C) DDFS in high genetic risk EBC population (HER2 low vs. HER2 0) [file DataSheet_1.zip › Supplementary Tables and Figures/Supplementary Table 2.docx]

Supplementary Table 2. Participant and Study Characteristics of Included Studies

| Source | Included sample size | Location | Median age, years | Median follow-up, years | Tumor size | Lymph node | Tumor grade | Stage | Data extraction tool |
| --- | --- | --- | --- | --- | --- | --- | --- | --- | --- |
| Carsten Denkert et al,^17^ 2021 | Total 2310  HR+1162  TNBC1148 | Europe | 48.5 | 3.9 | T1 36.5% T2 51.3% T3 7.6% T4a-c 2.5% T4d 1.7% Missing 0.4% | N0 62.1% N1 31.2% N2 3.9% N3 1.4% Missing 1.4% | G1 1.7% G2 32.0% G3 66.3% | NA | Didn’t use |
| Camille Domergue et al,^18^ 2022 | Total 437  HR+ 0  TNBC 437 | Europe | 51.0 | 6.1 | T0-T1 6.9% T2 55.8% T3 24.5% T4 12.8% | N0 39.4% N+ 60.6% | G2 24.0% G3 76.0% | NA | Used for partial data |
| Ryan Shea Ying Cong Tan et al,^19^ 2022 | Total 28280  HR+ 23503  TNBC 4634 | Asia | NA | 6.6 | T1 55.3% T2 37.6% T3 4.5% T4 1.7% Others 0.6% Unknown 0.3% | N0 64.0% N1 24.6% N2 7.1% N3 4.2% Unknown 0.1% | G1 22.4% G2 42.9% G3 28.6% Unknown 6.0% | Stage 1 42.4% Stage 2 42.6% Stage 3 15.0% | Didn’t use |
| George Douganiotis et al,^20^ 2022 | Total 949  HR+ 949  TNBC 0 | Europe | 51.0 | 2.8 | NA | NA | (For IDC only)  G1 26.9%  G2 44.6%  G3 27.9%  Unknown 0.6% | NA | Didn’t use |

Table 1. Participant and Study Characteristics of Included Studies (continued)

| Source | Included sample size | Location | Median age, years | Median follow-up, years | Tumor size | Lymph node | Tumor grade | Stage | Data extraction tool |
| --- | --- | --- | --- | --- | --- | --- | --- | --- | --- |
| Christopher Rosso et al,^21^ 2022 | Total 391  HoR+ 351  TNBC 38 | North America | 66.1 | 3.1 | NA | NA | G1 19.3% G2 48.2% G3 32.5% | Stage 1 50.4% Stage 2 35.8% Stage 3 7.2% Stage 4 6.6% | Didn’t use |
| Nanae Horisawa et al,^22^ 2022 | Total 4007  HoR+ 3541  TNBC 466 | Asia | NA | 5.0 | Tis 16.6% T<2cm 61.0% T: 2-5cm 18.7% T>5cm 2.6% Unknown 1.1% | N0 62.3% N1 17.8% N2-3 7.9% Unknown 12.0% | G1 30.9% G2 41.2% G3 18.1% Unknown 9.7% | NA | Didn’t use |
| Luciana de Moura Leite et al,^23^ 2021 | Total 855  HoR+ 542  TNBC 313 | South America | 45.3 | 4.9 | T1 3.3% T2 37.2% T3 29.6% T4 29.9% | N0 31.7% N1 45.6% N2 15.9% N3 6.8% | G1 5.8% G2 47.1% G3 43.0% Missing 4.0% | Stage 1 1.5% Stage 2 39.9%  Stage 3 58.6% | Used for partial data |
| Raz Mutai et al,^24^ 2021 | Total 608  HoR+ 608  TNBC 0 | Asia | 60.5 | 10.3 | T≤2cm 77.0% T>2cm 22.9%  Missing 0.1% | N0 82.6% | Low 14.0% Intermediate 53.9% High14.6%  Missing 17.5% | NA | Used for partial data |
| William Jacot et al,^25^ 2021 | Total 296  HoR+ 106  TNBC 187 | Europe | 57.7 | 9.7 | T1 45.6% T2 47.0% T3-T4 7.1% Missing 0.3% | N0 63.5% N+ 36.5% | G1-2 20.6% G3 77.4%  Missing 2.0% | NA | Didn’t use |

Table 1. Participant and Study Characteristics of Included Studies (continued)

| Source | Included sample size | Location | Median age, years | Median follow-up, years | Tumor size | Lymph node | Tumor grade | Stage | Data extraction tool |
| --- | --- | --- | --- | --- | --- | --- | --- | --- | --- |
| Paolo Tarantino et al,^26^ 2022 | Total^a^ 5235  HoR+ 4538  TNBC 697 | Multi-  continents | 59 | 0.8 | NA | N0 64.2% N+ 35.8% Unknown 0.0% | G1 28.7% G2 44.2% G3 25.9% Unknown 1.2% | Stage 1 58.7% Stage 2 17.9% Stage 3 2.9% Stage 4 20.5% | Didn’t use |
| Mengdi Chen et al,^27^ 2022 | Total 2099  HoR+ 2099  TNBC 0 | Asia | NA | 4.2 | T1 70.4% T2-4 29.6% | N0 83.9% N1-2 16.1% | G1 8.7% G2 57.2% G3 18.5% Unknown 15.6% | NA | Didn’t use |
| Han  gcheng Xu et al,^28^ 2022 | Total 777  HoR+ 678  TNBC 99 | Asia | 50 | 6.5 | T0/is/1 62.7% T2 34.4% T3 1.9% T4 0.5% Unknown 0.5% | N0 55.7% N1 27.0% N2 10.2% N3 6.6% Unknown 0.5% | G1 12.0% G2 59.1% G3 22.3% Unknown 6.7% | Stage 1 41.2% Stage 2 40.7% Stage 3 17.2% Unknown 0.9% | Didn’t use |
| Min Hwan Kim et al,^29^ 2020 | Total 2657  HoR+ 2657  TNBC 0 | Asia | 50 | 5.9 | T1 72.3% T2 26.2% T3 1.3% T4 0.1% | N0 67.7% N1 24.5% N2 6.1% N3 1.7% | G1 33.1% G2 56.9% G3 10.0% | Stage 1 55.7% Stage 2 36.0% Stage 3 8.3% | Didn’t use |
| Louis Fehrenbacher et al,^30^ 2019 | Total 3205^b^ | Multi-  continents | NA | 3.8 | NA | N0 21.5% N1 52.4% N2 18.0% N3 8.1% | Low 7.9% Intermediate 43.3% High 48.6% Unknown 0.2% | NA | Didn’t use |

Abbreviations: HoR+, hormone receptor-positive BC; NA, not available; N+, Lymph node positive.

a. Total population includes hormone receptor-positive BC and TNBC.

b. The number of patients included for the OS analysis in the study was 3205, though the overall population in the study was more than 3205.
