# Supplementary figures and images for: Clinicopathologic characteristics and prognostic significance of HER2-low expression in patients with early breast cancer: A systematic review and meta-analysis
Source: Front Oncol. 2023 Feb 2;13:1100332. doi: 10.3389/fonc.2023.1100332 (PMC9931719; doi:10.3389/fonc.2023.1100332)

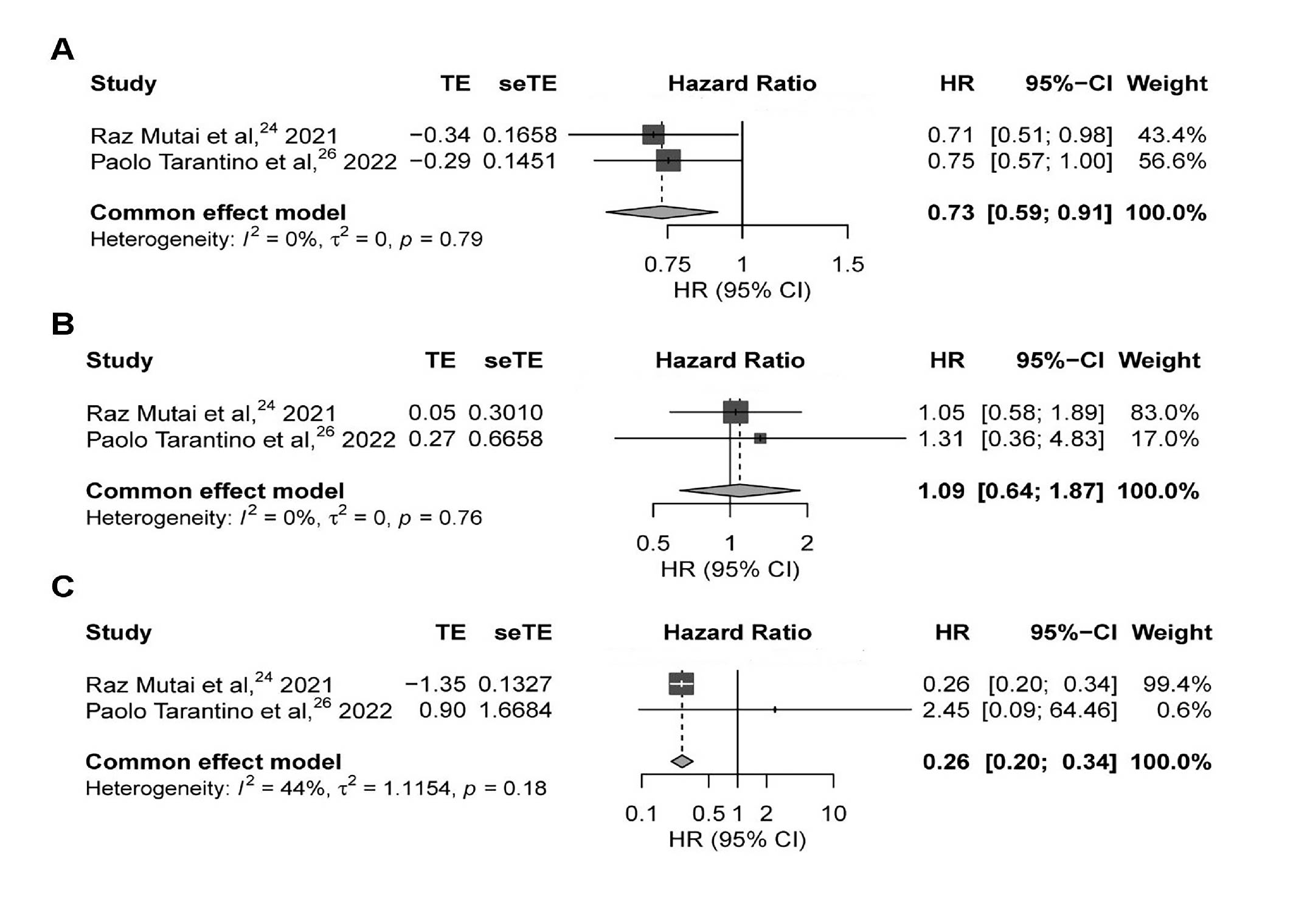

Supplement: Supplementary Figure 1 — Forest plot of (A) DDFS in hormone receptor positive subgroup (HER2 low vs. HER2 0) (B) DDFS in low genetic risk EBC population (HER2 low vs. HER2 0) (C) DDFS in high genetic risk EBC population (HER2 low vs. HER2 0) [file DataSheet_1.zip › Supplementary Tables and Figures/Supplementary Figure 1.jpeg]

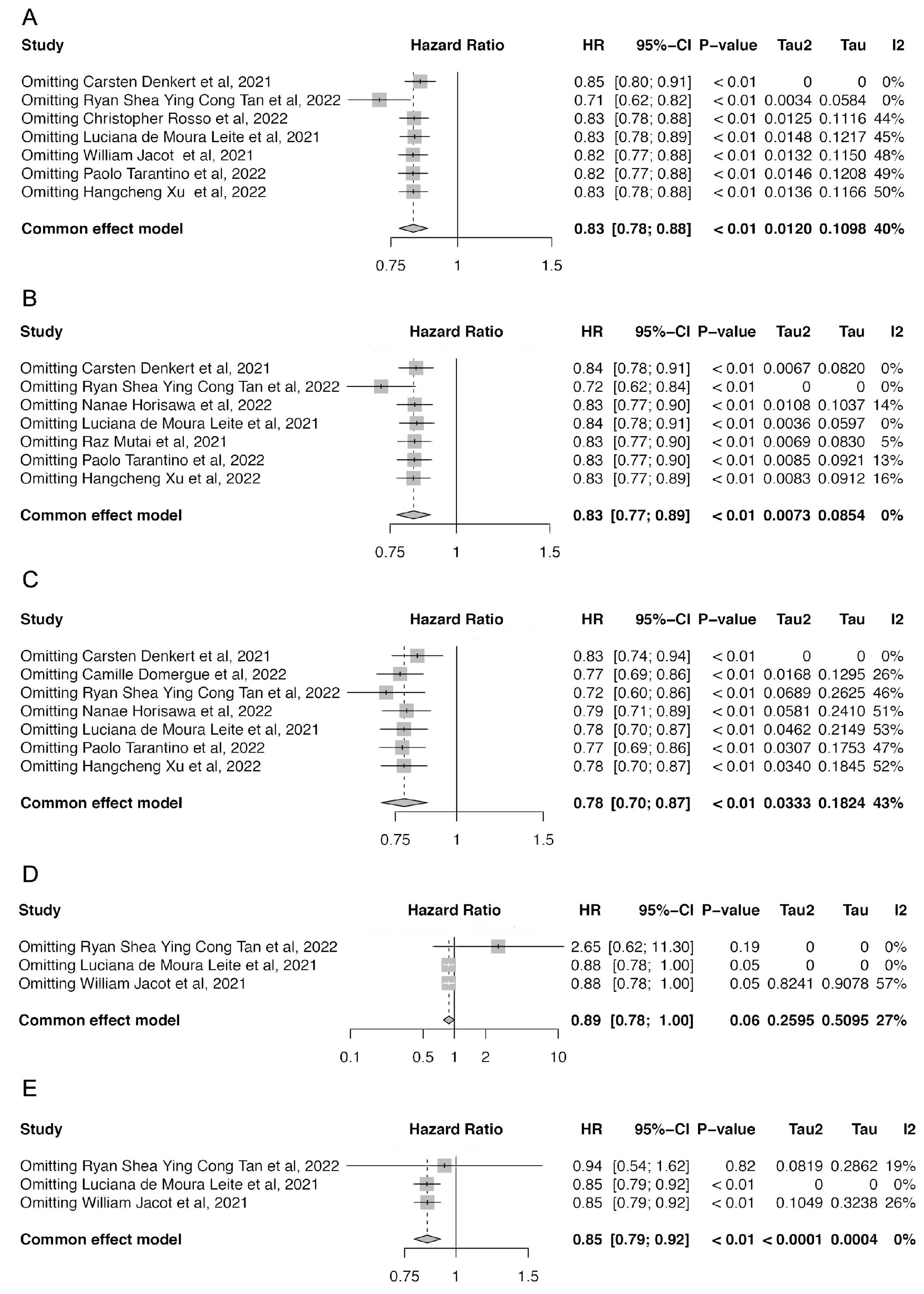

Supplement: Supplementary Figure 1 — Forest plot of (A) DDFS in hormone receptor positive subgroup (HER2 low vs. HER2 0) (B) DDFS in low genetic risk EBC population (HER2 low vs. HER2 0) (C) DDFS in high genetic risk EBC population (HER2 low vs. HER2 0) [file DataSheet_1.zip › Supplementary Tables and Figures/Supplementary Figure 2.jpeg]

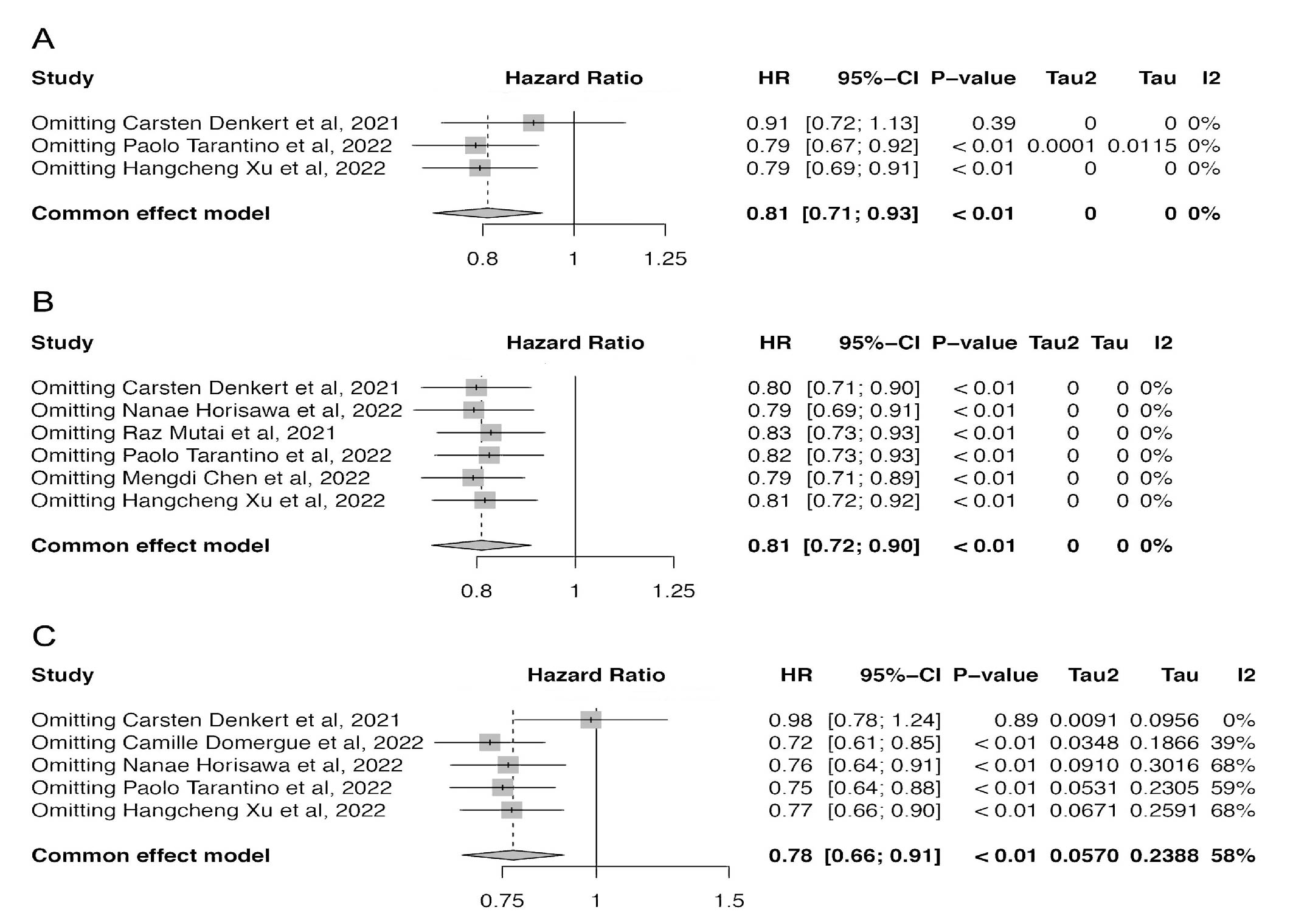

Supplement: Supplementary Figure 1 — Forest plot of (A) DDFS in hormone receptor positive subgroup (HER2 low vs. HER2 0) (B) DDFS in low genetic risk EBC population (HER2 low vs. HER2 0) (C) DDFS in high genetic risk EBC population (HER2 low vs. HER2 0) [file DataSheet_1.zip › Supplementary Tables and Figures/Supplementary Figure 3.jpeg]

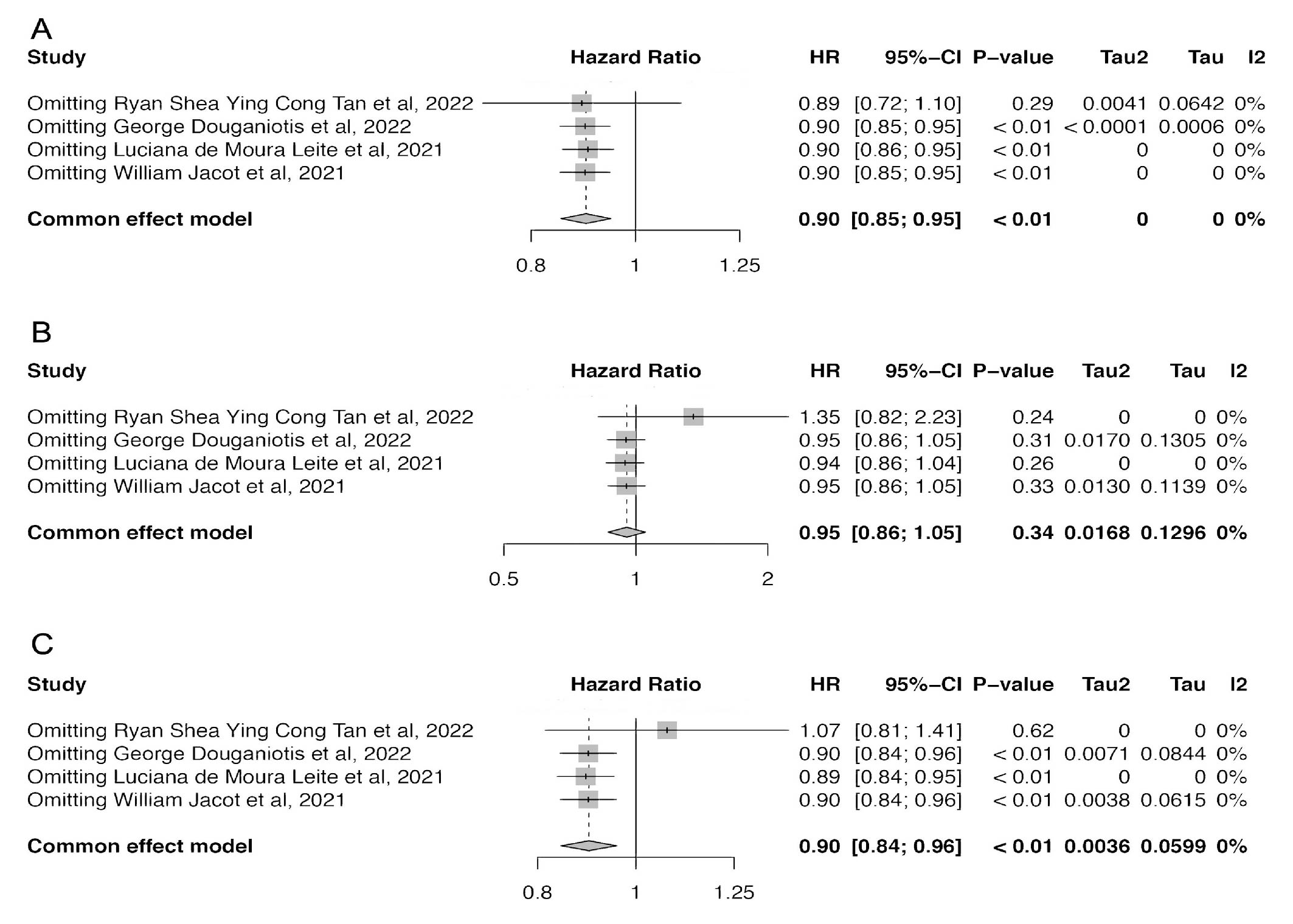

Supplement: Supplementary Figure 1 — Forest plot of (A) DDFS in hormone receptor positive subgroup (HER2 low vs. HER2 0) (B) DDFS in low genetic risk EBC population (HER2 low vs. HER2 0) (C) DDFS in high genetic risk EBC population (HER2 low vs. HER2 0) [file DataSheet_1.zip › Supplementary Tables and Figures/Supplementary Figure 4.jpeg]

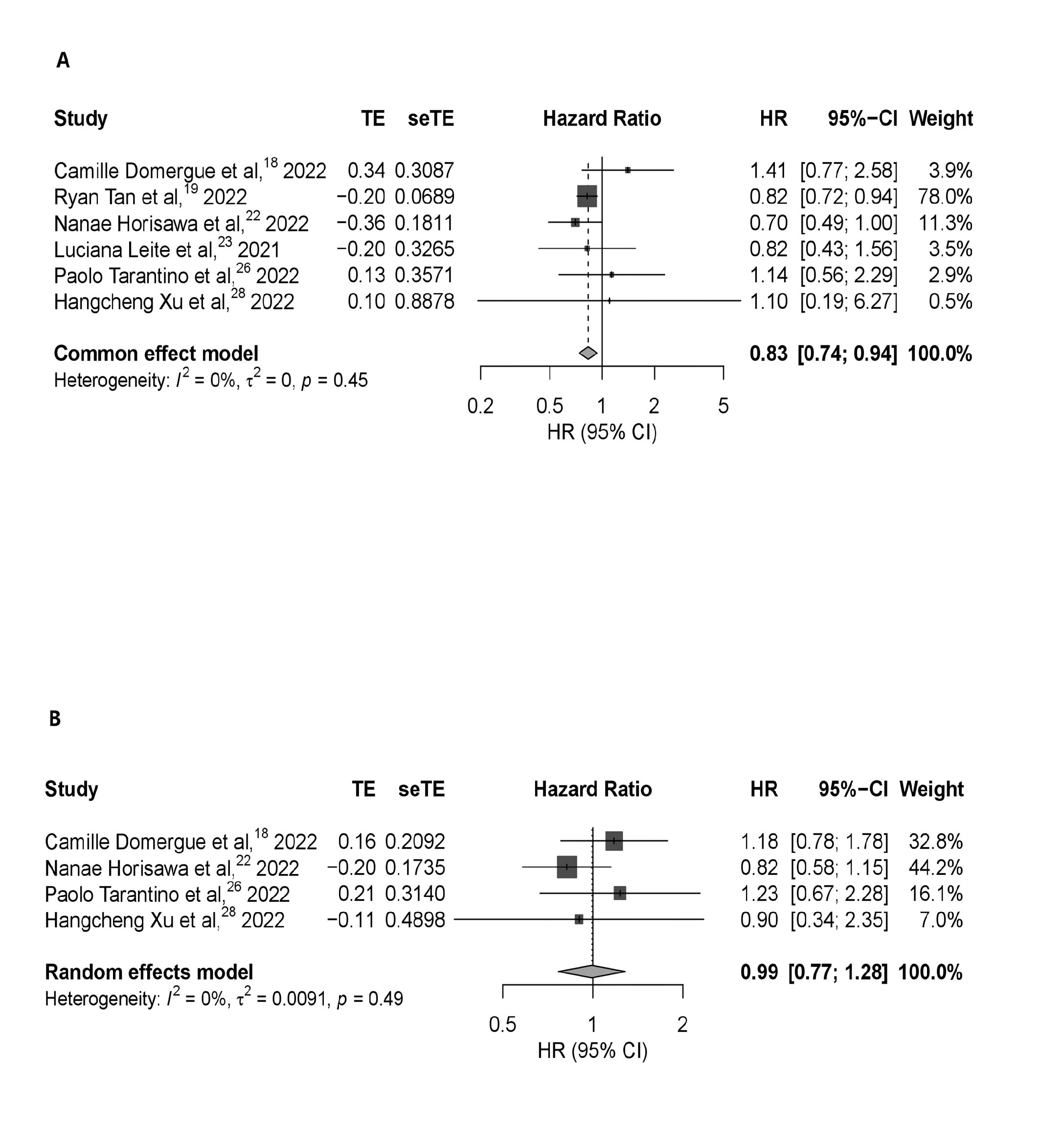

Supplement: Supplementary Figure 1 — Forest plot of (A) DDFS in hormone receptor positive subgroup (HER2 low vs. HER2 0) (B) DDFS in low genetic risk EBC population (HER2 low vs. HER2 0) (C) DDFS in high genetic risk EBC population (HER2 low vs. HER2 0) [file DataSheet_1.zip › Supplementary Tables and Figures/Supplementary Figure 5.jpeg]

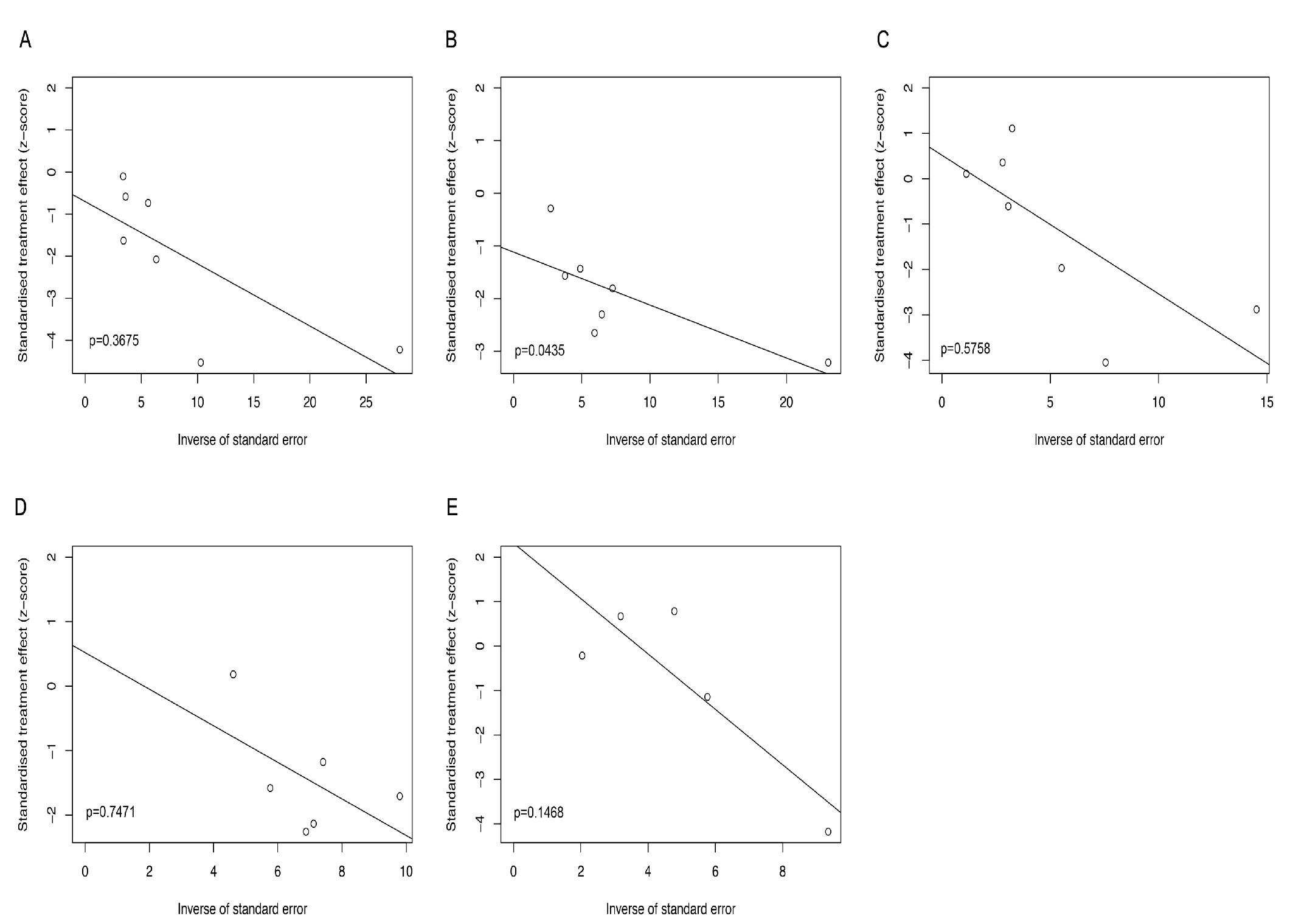

Supplement: Supplementary Figure 1 — Forest plot of (A) DDFS in hormone receptor positive subgroup (HER2 low vs. HER2 0) (B) DDFS in low genetic risk EBC population (HER2 low vs. HER2 0) (C) DDFS in high genetic risk EBC population (HER2 low vs. HER2 0) [file DataSheet_1.zip › Supplementary Tables and Figures/Supplementary Figure 6.jpeg]
