# Supplementary material for: Clinicopathologic characteristics and prognostic significance of HER2-low expression in patients with early breast cancer: A systematic review and meta-analysis
Source: Front Oncol. 2023 Feb 2;13:1100332. doi: 10.3389/fonc.2023.1100332 (PMC9931719; doi:10.3389/fonc.2023.1100332)
Supplement: Supplementary Figure 1 — Forest plot of (A) DDFS in hormone receptor positive subgroup (HER2 low vs. HER2 0) (B) DDFS in low genetic risk EBC population (HER2 low vs. HER2 0) (C) DDFS in high genetic risk EBC population (HER2 low vs. HER2 0) [file DataSheet_1.zip › Supplementary Tables and Figures/Supplementary Table 3. NOS.doc]

Supplementary Table 3. Newcastle - Ottawa Quality Assessment Scale for the included studies

| **Study** | **Selection** | | | | **Comparability** | **Outcome** | | | **Quality Score** |
| --- | --- | --- | --- | --- | --- | --- | --- | --- | --- |
|  | **Representativeness of the exposed cohort** | **Selection of the non exposed cohort** | **Ascertainment of exposure** | **Demonstration that outcome of interest was not present at start of study** | **Comparability of cohorts on the basis of the design or analysis** | **Assessment of outcome** | **Was follow-up long enough for outcomes to occur** | **Adequacy of follow up of cohorts** |  |
| Carsten Denkert et al,^17^ 2021 | ★ | ★ | ★ | ★ | ★ | ★ | ★ | ★ | 8 |
| Camille Domergue et al,^18^ 2022 | ★ | ★ | ★ | ★ | ★ | ★ | ★ | ★ | 8 |
| Ryan Shea Ying Cong Tan et al,^19^ 2022 | ★ | ★ | ★ | ★ | ★ | ★ | ★ | ★ | 8 |
| George Douganiotis et al,^20^ 2022 | ★ | ★ | ★ | ★ | ★ | ★ | ★ | ★ | 8 |
| Christopher Rosso et al,^21^ 2022 |  |  | ★ | ★ | ★ | ★ | ★ | ★ | 6 |
| Nanae Horisawa et al,^22^ 2022 | ★ | ★ | ★ | ★ | ★ | ★ | ★ | ★ | 8 |
| Luciana de Moura Leite et al,^23^ 2021 | ★ | ★ | ★ | ★ | ★ | ★ | ★ | ★ | 8 |
| Raz Mutai et al,^24^ 2021 | ★ | ★ | ★ | ★ | ★ | ★ | ★ | ★ | 8 |
| William Jacot et al,^25^ 2021 | ★ | ★ | ★ | ★ | ★ | ★ | ★ | ★ | 8 |
| Paolo Tarantino et al,^26^ 2022 | ★ | ★ | ★ | ★ | ★ | ★ |  | ★ | 7 |
| Mengdi Chen et al,^27^ 2022 | ★ | ★ | ★ | ★ | ★ | ★ | ★ | ★ | 8 |
| Han  gcheng Xu et al,^28^ 2022 | ★ | ★ | ★ | ★ | ★★ | ★ | ★ | ★ | 9 |
| Min Hwan Kim et al,^29^ 2020 | ★ | ★ | ★ | ★ | ★ | ★ | ★ | ★ | 8 |
| Louis Fehrenbacher et al,^30^ 2019 |  |  | ★ | ★ | ★ | ★ | ★ | ★ | 6 |
